# Harvesting Electricity with *Geobacter bremensis* Isolated from Compost

**DOI:** 10.1371/journal.pone.0034216

**Published:** 2012-03-28

**Authors:** Olivier Nercessian, Sandrine Parot, Marie-Line Délia, Alain Bergel, Wafa Achouak

**Affiliations:** 1 CEA, DSV, IBEB, SBVME, Lab Ecol Microb Rhizosphere and Environ Extrem (LEMiRE), Saint-Paul-lez-Durance, France; 2 CNRS UMR 7265 and FR CNRS 3098 ECCOREV, Saint-Paul-lez-Durance, France; 3 Aix-Marseille Université, Saint-Paul-lez-Durance, France; 4 Laboratoire de Génie Chimique, Centre National de la Recherche Scientifique, Université de Toulouse (INPT), Toulouse, France; Laurentian University, Canada

## Abstract

Electrochemically active (EA) biofilms were formed on metallic dimensionally stable anode-type electrode (DSA), embedded in garden compost and polarized at +0.50 V/SCE. Analysis of 16S rRNA gene libraries revealed that biofilms were heavily enriched in *Deltaproteobacteria* in comparison to control biofilms formed on non-polarized electrodes, which were preferentially composed of *Gammaproteobacteria* and *Firmicutes*. Among *Deltaproteobacteria*, sequences affiliated with *Pelobacter* and *Geobacter* genera were identified. A bacterial consortium was cultivated, in which 25 isolates were identified as *Geobacter bremensis*. Pure cultures of 4 different *G. bremensis* isolates gave higher current densities (1400 mA/m^2^ on DSA, 2490 mA/m^2^ on graphite) than the original multi-species biofilms (in average 300 mA/m^2^ on DSA) and the *G. bremensis* DSM type strain (100–300 A/m^2^ on DSA; 2485 mA/m^2^ on graphite). FISH analysis confirmed that *G. bremensis* represented a minor fraction in the original EA biofilm, in which species related to *Pelobacter* genus were predominant. The *Pelobacter* type strain did not show EA capacity, which can explain the lower performance of the multi-species biofilms. These results stressed the great interest of extracting and culturing pure EA strains from wild EA biofilms to improve the current density provided by microbial anodes.

## Introduction

Since their discovery [1]–[3] the interest towards electro-active (EA) microorganisms (also called exoelectrogenic bacteria or anode respiring bacteria) has been increasing, particularly because of their implication in microbial fuel cells (MFCs). Comprehensive reviews have been published on exoelectrogenic strains [4] and on the different electron transfer pathways between anodes and bacterial cells [5]. It has generally been found that, when tested in identical MFC devices, individual strains generate less power than mixed communities [4]. It is difficult so far to give definitive explanations. For example, comparisons made in MFCs equipped with an air-cathode may be detrimental to individual anaerobic strains because of the possible presence of oxygen traces in the anode compartment. Lower performance of individual strains may also indicate synergetic effects in multi-species biofilms [6] or may also be due to lower intrinsic efficiency of the strains that have been used until now to investigate mono-species microbial anodes.

Most studies devoted to single EA species have been carried out with type strains that corresponded to predominant species identified in wild EA biofilms. Thus far, only a few strains of bacteria have been directly isolated from EA biofilms [7], [8]. Nevertheless, when EA isolates have been compared to the corresponding type strain, they have shown promising electrochemical properties: *Ochrabactrum anthropi* isolates have given 89 mW/m^2^, while the type strain provided only 45 mW/m^2^ in identical conditions [7]. Nevertheless, in this case the power density provided by the pure culture remained lower than the power density provided by the original multi-species biofilm (539 mW/m^2^). In contrast, *Rhodopseudomonas palustris* isolated from a MFC has produced 56% larger power (2720 mW/m^2^) than the original biofilm (1740 mW/m^2^) [8]. These examples stressed the interest to work with EA isolates instead of type strains or non-controlled multi-species biofilms.

In this framework, a new procedure has recently been proposed in the literature to enlarge the possibilities of isolation of EA strains. It consisted of a two-chamber U-tube MFC coupled to successive dilution-to-extinction steps [7]. The procedure presented many advantages: i) it allowed bacteria to directly settle on the electrode surface and avoided thus the plating steps, which can grow only bacteria that are able to use soluble electron acceptors; ii) hexacyanoferrate(IV) used in the cathode compartment under nitrogen bubbling protected the anode from oxygen traces; iii) the hexacyanoferrate(IV)-reducing cathode allowed higher potential values than the conventional oxygen-reducing cathodes. Actually, it is admitted that forming EA biofilms from complex inoculum under polarization at low potentials results in strong selection, while the biofilms formed under higher potential polarization are more diverse [9]. It is consequently an advantage to use high potential, when the objective is to widely screen and identify as many EA strains as possible.

The purpose of this study was to identify the microbial community and then isolate and test in pure culture EA species that are responsible for harvesting electricity in biofilms formed from garden compost [10]. Anodes embedded in garden compost have been shown to develop EA biofilms that can produce up to 385 mA/m^2^ of current without the addition of acetate to the anode chamber [11]. However, analysis of the microbial community associated with these biofilms has not yet been studied. In order to perform as wide as possible microbial screening, the original biofilms were formed under polarization at high potential (+0.5 V/SCE). This potential has been identified as the maximum value that results high current values and reproducible current-time curves [12]. A three-electrode system associated to a potentiostat was used in the present study in order to reach this high potential value and to control it perfectly on long-term experiments. The microbial communities that formed EA biofilms were identified and compared by denaturing gradient gel electrophoresis to control biofilms formed without polarization. Isolates were extracted, cultured and their electrochemical capability tested with single-species biofilms. Fluorescence in situ hybridization was implemented to check the presence of the isolates in the original biofilms

## Results and Discussion

### Microbial consortia on polarized (PE) and non-polarized control (NPE) electrodes

Twelve Dimensionally Stable Anode (DSA) electrodes were embedded in garden compost that contained acetate (10 mM) used as electron donor. Six electrodes were individually polarized at 0.50 V/SCE and 6 control electrodes were not polarized. After one day, the current gradually increased due to the formation of an electrochemically active biofilm on the electrode surface, as previously reported [11], [12]. On 9^th^ day, when the current densities stabilized and averaged 300 mA/m^2^, the electrodes were removed to analyse the microbial communities attached on both polarized and control electrodes. Suspensions of microbial consortia obtained by scrapping were characterized by, total cell count of DAPI-stained cells, enumeration of cultivable heterotrophic bacteria and DNA gel quantification. The polarized electrodes (PE) were covered by a more important biofilm than the non polarized electrodes (NPE) as indicated by the higher amount of DNA extracted from biofilms formed on PE and also by a tenfold increase in the number of cultivable bacteria recovered from the latter biofilms. (Figure S1).

**Figure 1:**
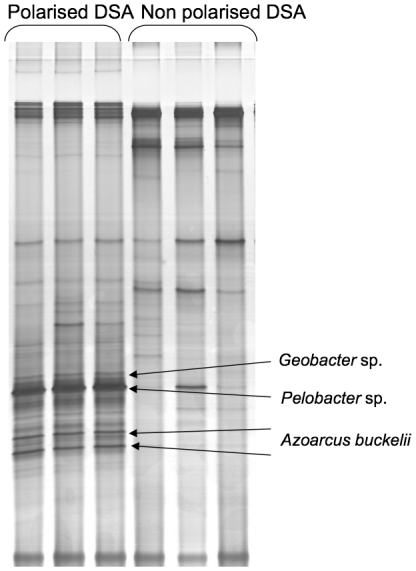
Impact of electrodes polarisation on bacterial community structure. Comparison of genetic fingerprints of compost EAB obtained on polarized (PE) and non-polarized (NPE) DSA by DGGE.

DGGE analysis showed different microbial compositions of the biofilms collected from the PE and from the NPE (Figure 1). Some bands present on the DGGE patterns of the 6 PE were absent or much less intense than for the NPE, which indicated that specific microbial populations were enriched on the PE. 16S rRNA genes clones' libraries were constructed from DNA extracted from the PE and NPE biofilms and each clone analysed by DGGE. Of the 89 and 73 clones of the PE and NPE, 19 and 27 DGGE groups were identified respectively (Figure S2). The most frequent DGGE pattern in the PE library occurred 61 times, while the most frequent DGGE pattern in the NPE library occurred 9 times, further arguing for an enrichment of specific microbial populations on polarized electrodes.

**Figure 2:**
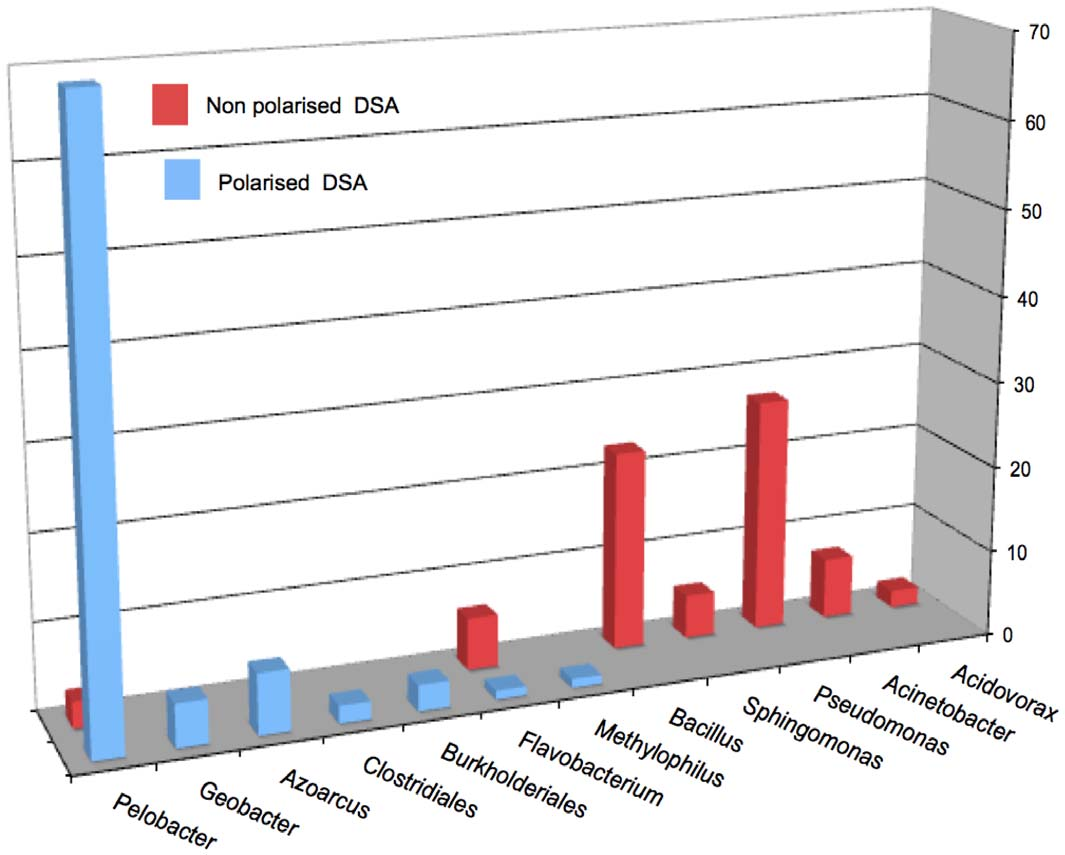
Identification of bacterial populations forming EAB. *rrs* based identification of clones from polarised and non polarised DSA clone libraries.

Analysis of 16S rRNA gene library revealed the presence of species related to *Deltaproteobacteria* and *Betaproteobacteria*. The PE were heavily enriched in *Deltaproteobacteria* in comparison to NPE that were preferentially colonized by *Gammaproteobacteria* and *Firmicutes*. The polarized electrodes showed mainly *Pelobacter, Geobacter, Azoarcus* and *Burkholderia*, whereas control electrodes were mainly colonized by bacteria related to *Pseudomonas*, *Bacillus*, *Sphingomonas*, *Acinetobacter* and *Acidovorax* (Figure 2). These data corroborated the identifications performed from DGGE bands (Figure 1). These results were consistent with previous studies reported for sediment MFCs that showed an enrichment of *Geobacteraceae* on current-harvesting electrodes compared to control electrodes that did not harvest power [13], [14]. Additionally, bacteria related to *Pelobacter* genus have also been described as major EA-biofilm-forming bacteria [15], [16].

The predominance of ethanol-fermenting bacteria primarily represented by the genus *Pelobacter* guided our investigation towards the use of ethanol and ferric-iron-based media for its cultivation under anaerobic conditions.

### Characterization of anaerobic dissimilatory ferric-iron-reducing bacteria

**Figure 3:**
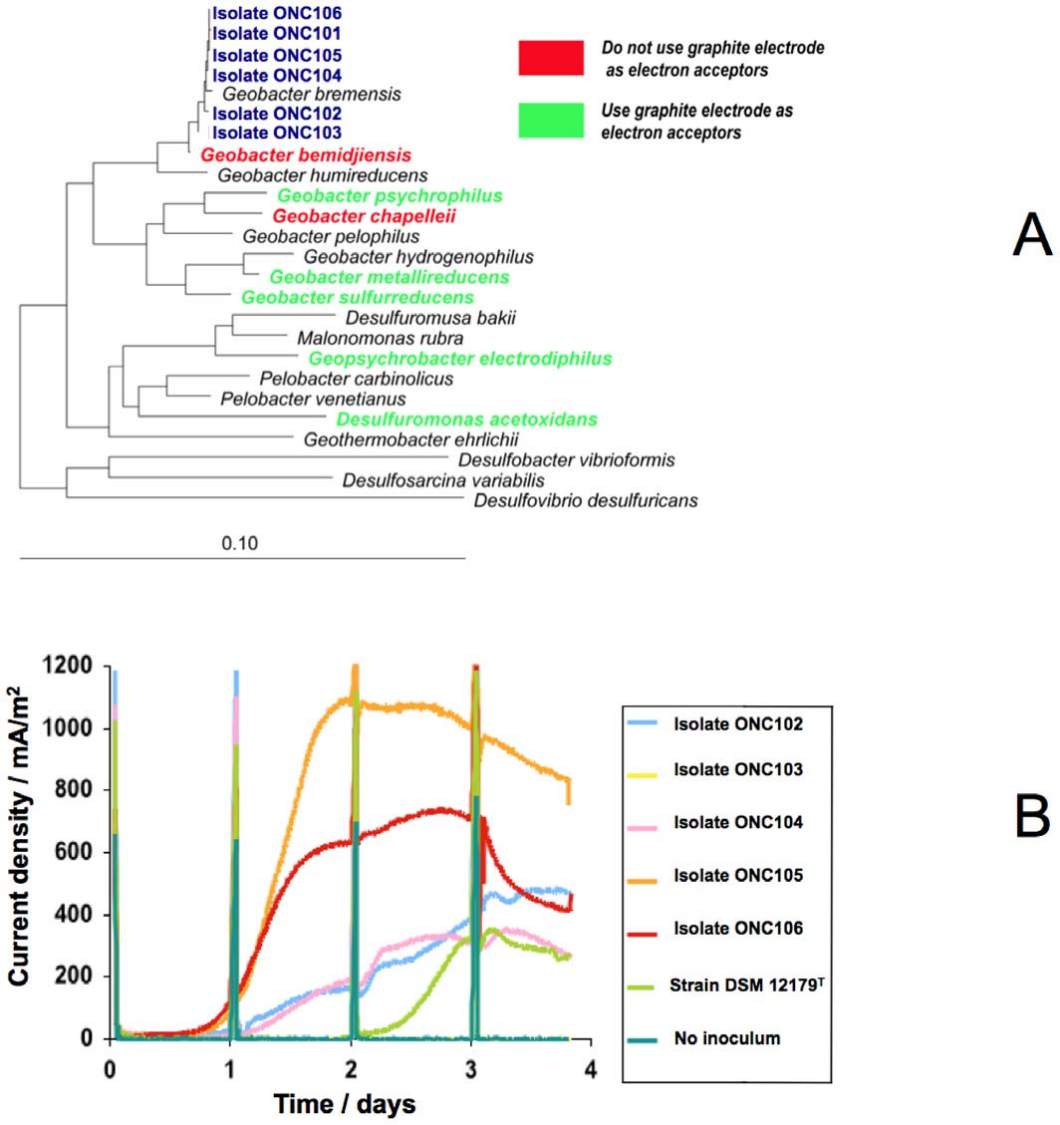
Phyloenetic and electrochemical activity of *G. bremensis* isolates. Phyloenetic tree based on *rrs* gene sequence (A) and chronoamerometry (B) of *G. bremensis* isolates and type strain DSM 12179.

Twenty five isolates were isolated from ethanol-iron oxide-based medium inoculated with a suspension of biofilm collected from the polarized electrodes. An affiliation of the 25 isolates with the family *Geobacteraceae* was revealed by comparative analysis of 16S rRNA gene sequences. Surprisingly, only bacteria related to *Geobacter* genus were isolated with approximately 99% sequence identity shared with *Geobacter bremensis* [17] (Figure 3A).

**Figure 4:**
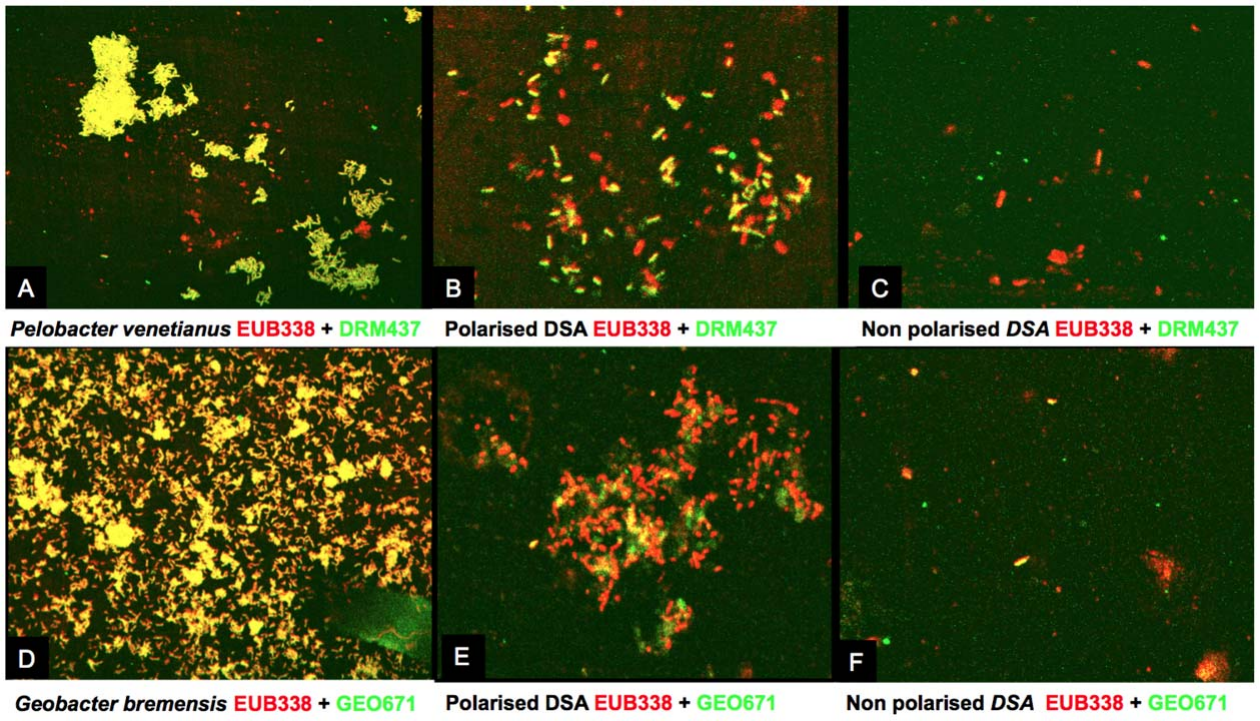
Localisation of *Pelobacter* and *Geobacter* species. FISH analysis of DSA electrodes by using 16 S rRNA probes directed specifically against *Pelobacter* species (ABC) and *Geobacter* species (DEF). Pictures A and D correspond to bacterial suspensions, B and E to polarised DSA, and C and F to non-polarised DSA.

Intra-species diversity within the 25 isolates was investigated by repetitive DNA-PCR (rep-PCR) fingerprinting [18]. The ERIC-PCR profiles revealed two clusters that differed from *G. bremensis* type strains (Figure S3). As both DGGE and 16S rRNA gene library data suggested that bacterial populations related to *Geobacter* genus were not predominant on PE compared to bacterial populations related to *Pelobacter*, we designed 16S rRNA probes directed specifically against *Pelobacter* species or against *Geobacter* species and used FISH technique to localize both bacterial genus on the original DSA electrodes retrieved from compost. FISH analysis confirmed that *Geobacter* represented a minor fraction of the original EA biofilm formed on polarized electrode, whereas *Pelobacter* was predominant. Both bacterial genera were not evidenced on NPE (Figure 4).

### Electrochemical activity of *Geobacter bremensis* isolates and type strain

Two isolates with ERIC profile 1, *G. bremensis* isolate ONC105 and isolate ONC106, two isolates with ERIC profile 2, *G. bremensis* isolate ONC102 and isolate ONC104, and the type strain DSM 12179 were cultured and used to inoculate (10% v/v) 5 different anaerobic reactors, which contained 10 mM ethanol and a DSA electrode polarized at 0.50 V vs. Ag/AgCl as the sole electron acceptor. In parallel, a control reactor was not inoculated. Current increase was observed in each reactor, except the control, showing after 3 days maximal values of 1100, 735, 485, 360 and 350 mA/m^2^ for isolates ONC106, ONC105, ONC102, ONC104 and the type strain, respectively (Figure 3B).

*Geobacter bremensis* is known to be able to transfer electrons to insoluble electron acceptors such as Fe(III) oxides [19], but to the best of our knowledge this was here the first time that *G. bremensis* strains were found to be able to transfer electrons to a solid electrode. Moreover, each isolate gave higher current density than the type strain and than the original multi-species biofilm. These results reinforced the few previous examples that indicated better performance of the isolates than the type strain [7] or than the original biofilm [8].

Several similar chronoamperometries performed with pure cultures of *Pelobacter venetianus* strain DSM 2395, which was related to *Pelobacter* spp. detected in the EA biofilms and run for more than 15 days did not exhibit any current production. Bacteria related to *Pelobacter* genus have already been described several times as major EA-biofilm-forming bacteria [15], [16], but it has also been reported that pure cultures have not shown any capability of producing current [20]. Here, DGGE, 16S rRNA gene library data and FISH imaging strongly confirmed that *Geobacter* genus represented a minor fraction of the original EA biofilm, whereas *Pelobacter* was predominant. Neither *Geobacter* nor *Pelobacter* genus were evidenced on control electrodes. Consequently, the development of *Pelobacter* spp. on the electrode surface was favoured by polarization, while pure cultures put in doubt its capacity to use the electrode as electron acceptor. It can be concluded that the enrichment of *Pelobacter* spp., which did not contribute to current harvesting, is a cause of the lower performance observed here for the original biofilms compared to the *Geobacter* pure cultures. It may be thought that *Pelobacter* related bacteria are non-EA bacteria that are enriched in EA biofilms and impediment the colonization by *Geobacter* spp.

### Comparison of DSA and graphite electrodes as electron acceptor

**Figure 5:**
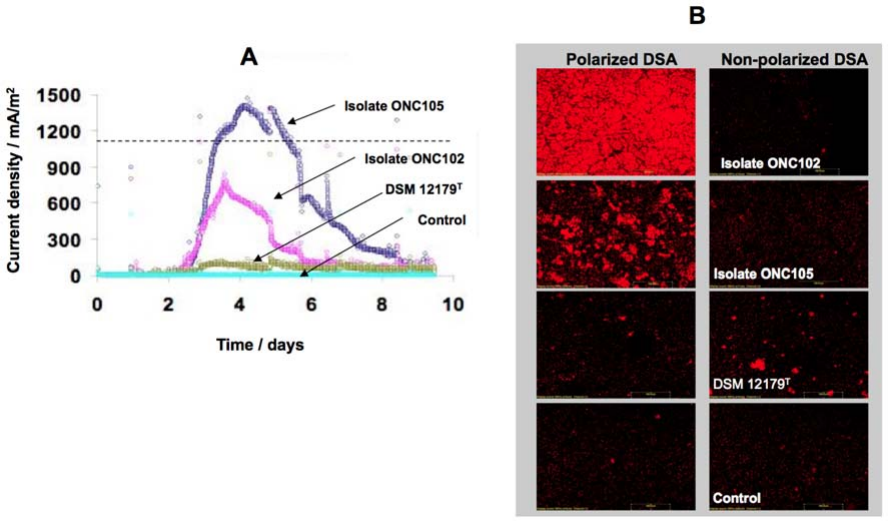
Localisation and electrochemical activity of *G. bremensis* isolates. Chronoamperometry (A) and FISH (B) performed with *G. bremensis* isolate ONC105, isolate ONC102, the type strain DSM 12179 and control experiment (not inoculated). A: Current density in 4 independent reactors with DSA electrodes polarized at 0.50 V vs Ag/AgCl as electron acceptor and ethanol (10 mM) as electron donor. B: FISH analysis of DSA electrodes by using 16 S rRNA probes directed specificaly against *Geobacter* species.

The isolates *G. bremensis* isolate ONC105 and isolate ONC102, which showed the highest current density for each ERIC profile, and the *G. bremensis* type strain were inoculated in similar anaerobic conditions (10 mM ethanol) in different reactors with DSA or graphite electrodes polarized at 0.50 V/SCE. On DSA electrodes, current increased from day 1.5 reaching maximal values of 1400, 750 and 100 mA/m^2^ for isolate ONC105, isolate ONC102 and type strain, respectively (Figure 5A). These values were slightly different from the previous series but gave the same ranking from isolate ONC105 with highest current density to the type strain with the lowest. Current production was nicely correlated to the ability of these isolates to colonize DSA electrodes as evidenced by FISH analysis (Figure 5B).

**Figure 6:**
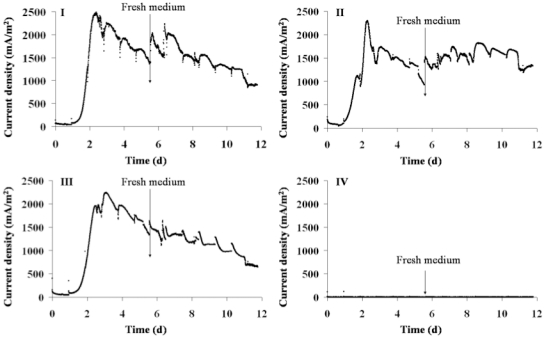
Current density increase in 4 independent reactors with graphite electrodes. Graphite electrodes were polarized at 0.50 V vs Ag/AgCl. Ethanol (10 mM) was used as electron donor. *G. bremensis* isolate ONC105 (Graph I), *G. bremensis* isolate ONC102 (Graph II), *G. bremensis* strain DSM 12179 (Graph III) and control experiment (Graph IV).

On graphite electrodes, current increased from day 1 and reached maximal values of 2485, 2290 and 2240 mA/m^2^ for *G. bremensis* isolate ONC105, isolate ONC102 and type strain, respectively (Figure 6). Graphite gave higher current density than DSA and showed less discrepancy between each strain. With each electrode, after reaching the maximal value the current density continuously decreased. For these experiments performed with graphite electrodes, the medium was replaced by fresh medium after 5 days, allowing the current density to stabilise with *G. bremensis* isolate ONC102 (Graph II in Figure 6). However, medium replacement had only a slight effect with isolate ONC105 and no effect with the type strain (Graph I and III in Figure 6).

**Figure 7:**
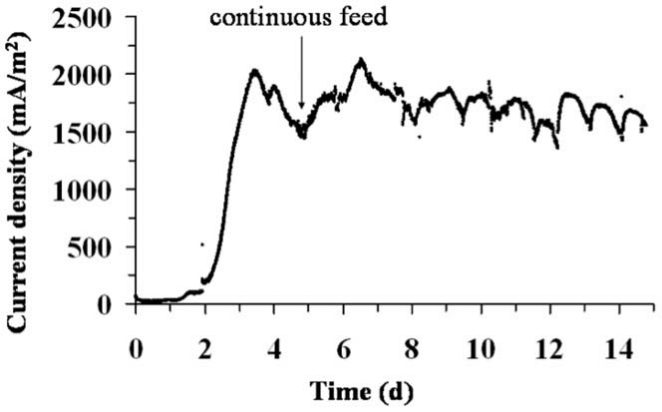
Chronoamperometry with *G. bremensis* isolate ONC105. Graphite electrode were polarized at 0.50 V vs Ag/AgCl. At 5th day, the reactor was fed continuously with fresh medium (residence time 30 h).

Finally, a reactor containing a graphite electrode polarized at 0.50 V vs Ag/AgCl was inoculated with *G. bremensis* isolate ONC105 in the same manner as previously described. The current density increased from day 1 until reaching 2035 mA/m^2^ (Figure 7). Since day 5, the reactor was then fed continuously with fresh medium with a residence time of 30 hours. It was thus succeeded in stabilizing the current density for more than 10 days in the range 1500 to 2100 mA/m^2^ (1730 mA/m^2^ in average), with circadian oscillation as commonly observed with EA biofilms [10].

The different strains of *Geobacter bremensis* that were extracted from wild EA biofilms revealed thus able to produce current density three-fold higher (up to 1400 mA/m^2^) than the original biofilms (average 300 mA/m^2^) in identical conditions (DSA electrode) and up to eight-fold higher when the electrode was changed to graphite (up to 2490 mA/m^2^ and around 1730 mA/m^2^ stable for days). This work is a supplementary example, among the few that have already been reported, of the great interest of extracting and culturing pure EA strains to improve the current density provided by microbial anodes.

## Materials and Methods

### Forming EA biofilms from garden compost

Twelve Dimensionally Stable Anode (DSA®, Electro Chemical Service) electrodes (50×25×1 mm) were cleaned and embedded in a 10 L tank containing garden compost mixed with 10 mM acetate and 10 mM NaCl as previously described [12]. DSA are titanium electrodes coated by iridium and tantalum oxides, which are specifically designed for industrial electrochemical processes. The three-electrode system that was implemented used a carbon auxiliary electrode with large surface area and a saturated calomel reference electrode (SCE, potential = 0.24 vs. SHE). Six electrodes were polarized at +0.50 V. Each electrode was individually connected to the same reference electrode and auxiliary electrode using a multi-channel potentiostat equipped with the suitable NStat system (VMP2 Bio-Logic SA). Six control electrodes were not connected to the electrical circuit.

### Cultivation procedures

Microbial suspensions obtained from the polarized and control DSA electrodes were used to inoculate the anthraquinone-2,6- disulfonate (AQDS) Freshwater basal medium, which was slightly modified and consisted in (in grams per litre of water) NH_4_Cl (0.25), NaH_2_PO_4_ (0.6), KCl (0.1), NaHCO_3_ (2.5), AQDS (0.515), 1 mL trace elements solution SL-10 and 1 mL vitamin solution (medium DSMZ 320). The Fe-citrate–Freshwater basal medium was prepared according to the medium DSMZ 579 with slight modification and consisted in (in grams per liter) FeIII-citrate (13.7), NaHCO_3_ (2.5), NH_4_Cl (1.5), NaH_2_PO_4_ (0.6), KCl (0.1), 1 mL trace elements solution SL-10, and 1 mL vitamin solution. Acetate (10 mM) and lactate (10 mM) were provided as carbon and electron sources in these two media. The Fe(III)-nitrilotriacetic acid (NTA))-Freshwater basal medium was prepared according to the medium DSMZ 298 with slight modification and consisted in (in grams per litre of water) NaH_2_PO_4_ (0.2), NH_4_Cl (0.25), NaCl (1), MgCl_2_. 6 H2O (0.4), KCl (0.5), CaCl_2_.2H_2_O (0.15), NaHCO_3_ (2.5), FeIII-NTA (5 mM), 1 mL trace elements solution SL-10, and 1 mL selenite/tungstate solution (medium DSMZ 385). Fe(III)-NTA was obtained from a 100 mM solution prepared in dissolving 1.64 g NaHCO_3_, 2.56 g C_6_H_6_NO_6_Na_3_ (sodium nitrilotriacetic acid) and 2.7 g FeCl_3_.6H_2_O in distilled water for a total volume of 100 mL. Solutions of Fe(III)-NTA, ethanol, K_2_HPO_4_, NaHCO_3_ were sterilized by filtration at 0.2 µm and added after autoclaving. Na_2_S was autoclaved separately. The medium was then dispensed into 50-mL sealed bottles and bubbled with N_2_/CO_2_ (80∶20) for 15 min. pH was 6.6, incubation was done at 30°C. A modified version of this media was also used and consisted in (in grams per litre of water): NH_4_Cl (1), MgSO_4_.7 H2O (0.2), CaCl_2_.2H_2_O (0.1), K_2_HPO_4_ (0.05), NaHCO_3_ (0.43), Fe(III)-NTA (5 mM), 1 mL trace elements solution SL-10, and 1 mL selenite/tungstate solution. Ethanol (10 mM) was provided as carbon and electron sources in these two media. Cultures were performed at 28°C in the dark. Positive cultures were obtained by three tenfold-serial dilutions, and were further purified by successive transfer in fresh medium. Finally, single colonies were obtained in agar-solidified media. The isolates were identified by sequencing the 16S rRNA gene. The type strains *Geobacter bremensis* (DSM 12179) and *Pelobacter venetianus* (DSM 2395) and isolates were grown in medium containing 5 mM Fe(III)-NTA as the electron acceptor and 10 mM ethanol as the electron donor and (in grams per litre of water) NH_4_Cl (1); (0.2) MgSO_4_.7H_2_O (0.2); CaCl_2_.2H_2_O (0.1); K_2_HPO_4_ (0.05); NaHCO_3_ (0.43); 50 µL of 10 mg/L resazurin; 1 mL of Na_2_S 10% (w/v); 1 mL element trace solution SL10 (DSM, Germany); and 1 mL of a selenite/tungstate solution (DSM, Germany).

### Electrochemical experiments with pure cultures

DSA (50×25×1 mm) and graphite (50×25×5 mm, Goodfellow) electrodes were cleaned as described elsewhere [21]. Electrodes were polarized at 0.50 V vs. Ag/AgCl using a potentiostat (VMP2 Bio-Logic SA) with a platinum grid as counter electrode and a silver/silver chloride as reference electrode. Before use, the platinum grid was red heated and the reference electrode was rinsed with ethanol. Pure cultures were inoculated in anaerobic reactors containing 500 mL medium with a 100-mL headspace. N_2_/CO_2_ (80∶20) was continuously bubbled in the medium through a 0.2-µm filter. All experiments were performed at 30°C. The reactors were filled with the medium used for bacterial growth that did not contain the electron acceptor Fe(III)-NTA, and Na_2_S (Na2S created current abiotically). Reactors were inoculated with 10% of culture solution, 24 hours after Fe(III)-(NTA) had been completely reduced to Fe(II), as indicated by the disappearance of the brown colour. Control experiments performed with the culture medium showed no current production in the absence of Fe(III)-(NTA). Cyclic voltammetry at 10 mV/s started from 0.50 V towards positive values between −0.80 and 0.80 V vs. Ag/AgCl with graphite electrodes.

### Cells recovery from biofilms and DNA extraction

Biofilms were recovered from electrodes as previously described [22], turbidity measurements were performed at OD_595_ to estimate bacterial suspensions from biofilms. DNA was extracted from biofilms according to Erable *et al.* [23].

### Construction of 16S rRNA genes libraries

DNA samples extracted from the 6 connected DSA were pooled. The entire 16S rDNA gene (*rrs*) amplification was performed using bacterial primers (fD1, rD1) as described previously by Achouak *et al.* [24]. The 16S rDNA fragments were ligated into Topo XL cloning vector (Invitrogen) according to the manufacturer's instructions. For the connected DSA and the control DSA, 89 and 75 colonies were randomly picked and stored at −80°C respectively. The *rrs* fragments were screened by denaturing gradient gel electrophoresis (DGGE), and those representing different DGGE patterns were sequenced.

### Denaturing gradient gel electrophoresis (DGGE) fingerprinting

16S rRNA genes fragments were amplified with primers P2 and P3 [25] and analysed by DGGE. Briefly, PCR products were loaded in a polyacrylamide gel containing a gradient of denaturant (32%–62%) and separated according to their GC content by electrophoresis (75 V, 60°C, 17 h). 16S rRNA genes fragments were sequenced as previously described [26].

### Genomic fingerprinting using ERIC-PCR

Cell lysis was carried out by heating 10 µL of the bacterial suspension at 95°C for 15 min in the PCR reaction buffer and then adding the enzyme. Amplification reactions were performed as described by Achouak *et al.* [27].

### *rrs* sequence analysis

The 16S rRNA genes sequences were submitted to the BLAST program of the National Center for Biotechnology Information [28] and to the Sequence Match of the Ribosomal Database Project [29] to identify the closest relatives. 16S rRNA genes sequences were subsequently imported in the software ARB [30] and aligned against their closest relatives using the program Integrated Aligner. Phylogenetic analyses were performed using the neighbour-joining method [31]. Short 16S rRNA genes sequences obtained from DGGE gels were added to the tree using the tool Add by Parsimony.

### Accession numbers

The sequences from this study were deposited to GenBank under the accession numbers JN795168-JN795237.

### Microscopy analysis of isolates on DSA

Microbial consortia suspensions scrapped from the polarized and control DSA electrodes were sub-sampled and fixed with 4% paraformaldehyde for 1.5 h at room temperature. Total cell counts of microbial suspension of consortia and isolates were determined with 4,6-diamino-2-phenylindole (DAPI) and the use of epifluorescence microscopy. Fluorescence In Situ Hybridization (FISH) was performed as previously described [32] using probes Cy5-EUB338, Fluoresceine-DRM437 [33], and the newly designed probe Fluoresceine-GEO671. The stringency conditions for hybridization with probes EUB338/DRM437 and EUB 338/GEO671 were evaluated in formamide gradients using reference strains as target and non-target cells. Hybridizations were conducted at 46°C for at least 2 h in 20% formamide hybridization buffer, with washing at 48°C. Biofilm coverage and architecture were determined by confocal scanning laser microscopy (CSLM).

## Supporting Information

Figure S1 **Impact of electrodes polarisation on biofilms formation.** Nucleic acids contents and cultivable bacterial cells number from polarized DSA (PE) and non-polarized DSA (PE) electrodes.
(TIF)

Figure S2 **Bacterial diversity of EAB.** Bacterial diversity of *rrs* clones library from polarised and non-polarised DSA, evaluated by number of different DGGE profile.
(TIF)

Figure S3 **Genotyping of** ***Geobacter sp.*** **isolates.** Selected isolates of *Geobacter sp.* ONC1001-1006 (lines A–F) and *G. bremensis* (Lline G) type strain DSM 12179 were characterised by ERIC-PCR.
(TIF)
