# Supplementary figures and images for: Harvesting Electricity with Geobacter bremensis Isolated from Compost
Source: PLoS One. 2012 Mar 28;7(3):e34216. doi: 10.1371/journal.pone.0034216 (PMC3314594; doi:10.1371/journal.pone.0034216)

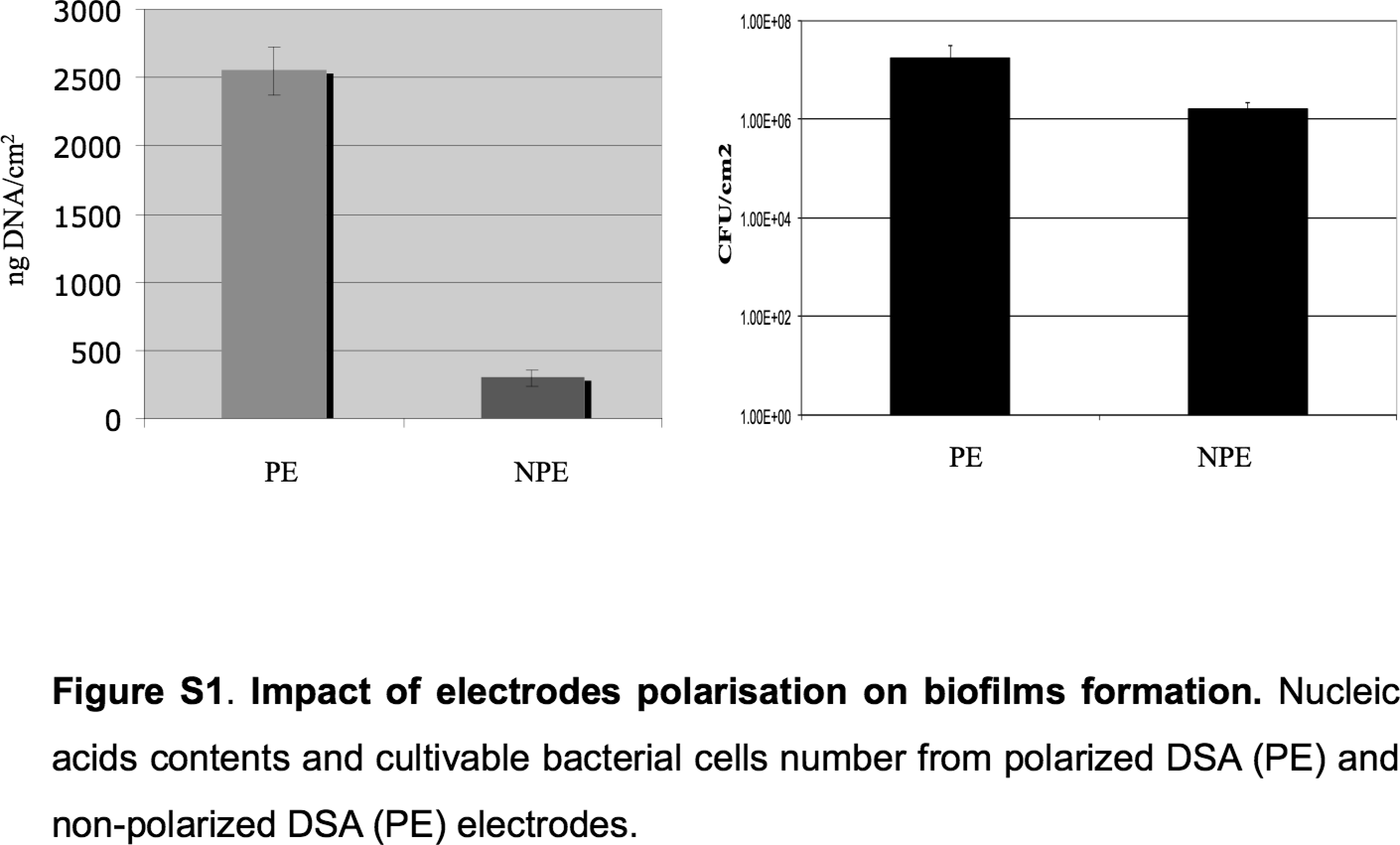

Supplement: Figure S1 — Impact of electrodes polarisation on biofilms formation. Nucleic acids contents and cultivable bacterial cells number from polarized DSA (PE) and non-polarized DSA (PE) electrodes. (TIF) [file pone.0034216.s001.tif]

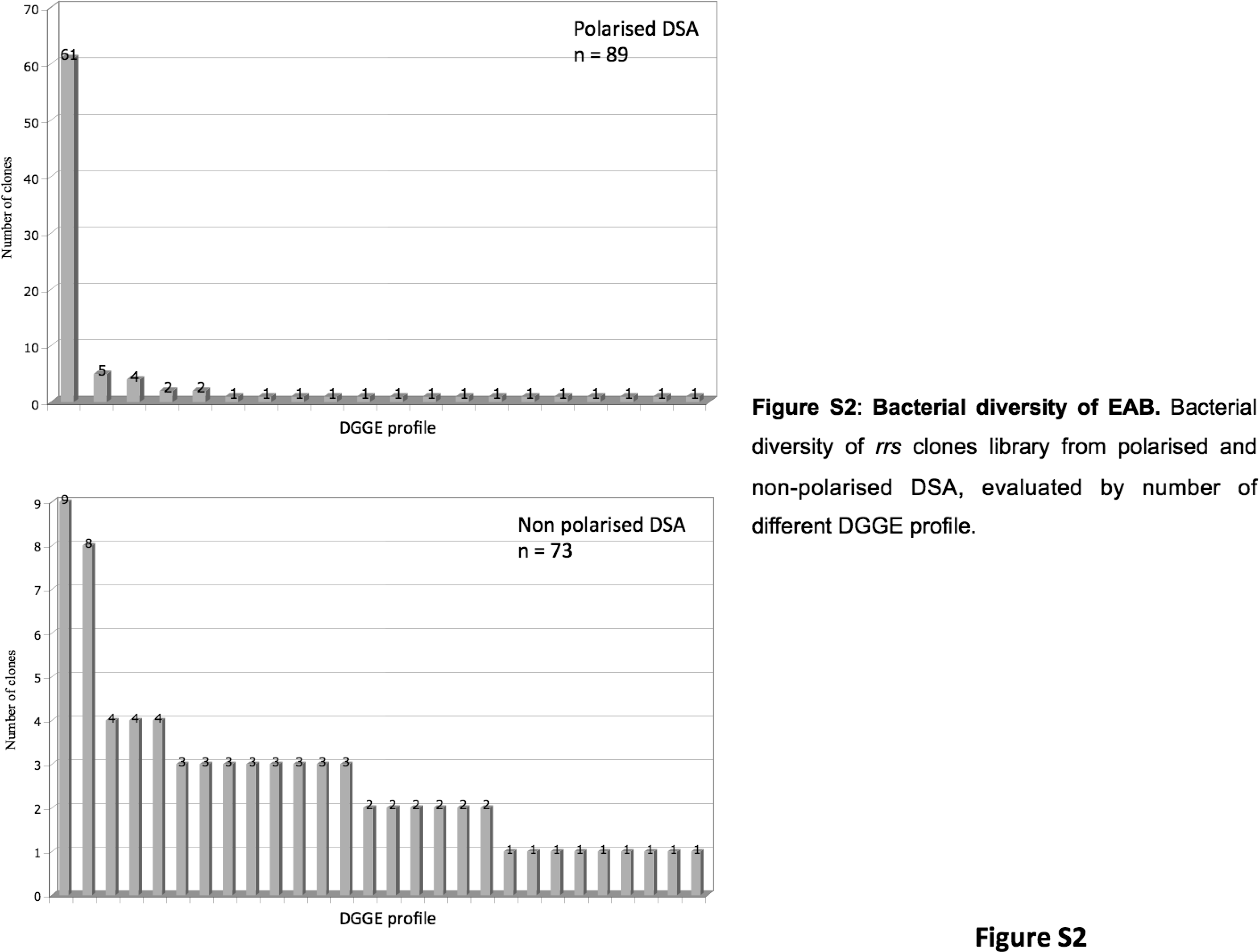

Supplement: Figure S2 — Bacterial diversity of EAB. Bacterial diversity of rrs clones library from polarised and non-polarised DSA, evaluated by number of different DGGE profile. (TIF) [file pone.0034216.s002.tif]

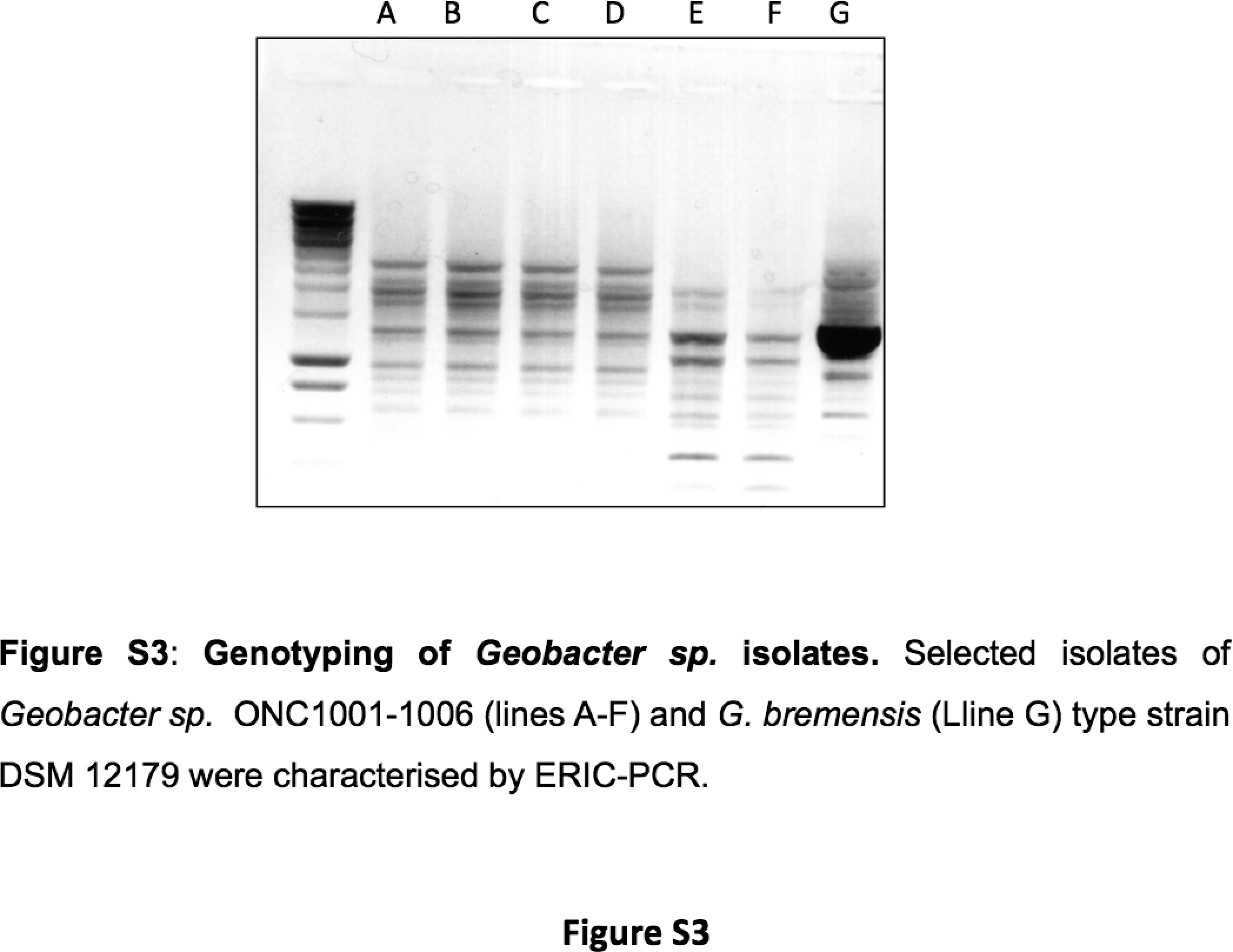

Supplement: Figure S3 — Genotyping of Geobacter sp. isolates. Selected isolates of Geobacter sp. ONC1001-1006 (lines A–F) and G. bremensis (Lline G) type strain DSM 12179 were characterised by ERIC-PCR. (TIF) [file pone.0034216.s003.tif]
